# Attentional Processes in Children With Attentional Problems or Reading Difficulties as Revealed Using Brain Event-Related Potentials and Their Source Localization

**DOI:** 10.3389/fnhum.2020.00160

**Published:** 2020-05-08

**Authors:** Praghajieeth Raajhen Santhana Gopalan, Otto Loberg, Kaisa Lohvansuu, Bruce McCandliss, Jarmo Hämäläinen, Paavo Leppänen

**Affiliations:** ^1^Department of Psychology, University of Jyväskylä, Jyväskylä, Finland; ^2^Graduate School of Education, Stanford University, Stanford, CA, United States

**Keywords:** attention, ANT, event-related potentials, N1, P3, source analysis, attentional problems, reading difficulties

## Abstract

Visual attention-related processes include three functional sub-processes: alerting, orienting, and inhibition. We examined these sub-processes using reaction times, event-related potentials (ERPs), and their neuronal source activations during the Attention Network Test (ANT) in control children, attentional problems (AP) children, and reading difficulties (RD) children. During the ANT, electroencephalography was measured using 128 electrodes on three groups of Finnish sixth-graders aged 12–13 years (control = 77; AP = 15; RD = 23). Participants were asked to detect the direction of a middle target fish within a group of five fish. The target stimulus was either preceded by a cue (center, double, or spatial), or without a cue, to manipulate the alerting and orienting sub-processes of attention. The direction of the target fish was either congruent or incongruent in relation to the flanker fish, thereby manipulating the inhibition sub-processes of attention. Reaction time performance showed no differences between groups in alerting, orienting, and inhibition effects. The group differences in ERPs were only found at the source level. Neuronal source analysis in the AP children revealed a larger alerting effect (double-cued vs. non-cued target stimuli) than control and RD children in the left occipital lobe. Control children showed a smaller orienting effect (spatially cued vs. center-cued target stimuli) in the left occipital lobe than AP and RD children. No group differences were found for the neuronal sources related to the inhibition effect. The neuronal activity differences related to sub-processes of attention in the AP and RD groups suggest different underlying mechanisms for attentional and reading problems.

## Introduction

Attentional problems (AP) and reading difficulties (RD) are two of the most common developmental problems that hinder learning in children (American Psychiatric Association, 2013). These difficulties increase the risk of serious academic, economic, and psychosocial consequences (de Kieviet et al., 2012; Sexton et al., 2012). Visual Attention Network Test (ANT) studies are increasingly used to understand these difficulties in typically and atypically developing children (Bednarek et al., 2004; Mezzacappa, 2004; Rueda et al., 2004a, 2012; Adólfsdóttir et al., 2008; Kratz et al., 2011; Mullane et al., 2011; Liu and Sun, 2017). However, there is a lack of neuronal-level brain information related to ANT in children with AP and children with RD in the same study. In this study, we examined reaction time (RT) performance, scalp topography of event-related potentials (ERPs), and their neuronal sources associated with attention network sub-processes using an Attention Network Test (ANT) (Santhana Gopalan et al., 2019) in AP and RD children.

Attention-deficit/hyperactivity disorder (ADHD) is a common childhood psychiatric disorder with a strong genetic and neurobiological basis (National Collaborating Centre for Mental Health (UK), 2018). The symptoms of AP defined here are similar to those of ADHD, and they include short attention span, excessive activity, and impulsivity (American Psychiatric Association, 2013). The bottom-up theories of neurobiology of ADHD propose disturbances in subcortical regions such as the thalamus, hypothalamus, and striatum. It has been suggested that these brain regions play an important role in ADHD groups during motor inhibition (Matthews et al., 2014; Singh et al., 2015). The top-down theories attribute dysfunction to frontal and prefrontal cortices (Singh et al., 2015). These regions seem to be associated with spatially focusing attention, resisting distractions, and developing an awareness of self and of time (Bellman, 2002, 104). Individuals with ADHD show deviant activation patterns in the anterior and frontal cortices (dorsolateral prefrontal cortex and orbitofrontal cortex) with greater involvement of the right hemisphere (Posner and Raichle, 1994) and parietal cortex (Konrad et al., 2006; Booth et al., 2007).

Reading difficulties, commonly referred to as dyslexia, are characterized by a number of difficulties (Wagner and Torgesen, 1987; Wimmer, 1993; Wagner et al., 1994; Feinberg and Farah, 2003, 802; Lyon et al., 2003; Vellutino et al., 2004; Wimmer and Schurz, 2010; Rose and Rouhani, 2012). Individuals with dyslexia are often considered poor readers despite their normal intelligence and adequate educational provision (Rutter and Yule, 1975; Lyon et al., 2003; Vellutino et al., 2004). Some studies have interpreted the impaired performance of dyslexic children in visual tasks as evidence of a deficit in visual processing (Facoetti et al., 2006, 2008; Bosse et al., 2007). In reading, the dorsal stream (occipito-parietal pathway) allocates attention to appropriate areas of text (spatial location), providing sufficient feedback to the ventral stream (occipito-temporal pathway) for processing or analysis of letters (Jones et al., 2008). A dorsal stream deficit might therefore impede smooth attentional focus on orthographic items, disrupting the visual recognition of letters that is accomplished by the ventral stream. The late stages of dorsal stream functioning involve the parietal cortex, which serves to deploy and control visual attention across different regions of the visual field. In line with these observations, individuals with dyslexia might have visual deficits that originate in the dorsal processing stream (Pammer and Vidyasagar, 2005). These findings suggest some common attentional deficits in RD and AP.

Previous studies involving random and clinical samples have consistently shown that ADHD and dyslexia overlap and are inter-related (Mayes et al., 2000; Carroll et al., 2005; Maughan and Carroll, 2006; Germanò et al., 2010). The overlap of these difficulties is described as co-occurring rather than involving comorbidity, which implies that the background factors are not causally related (Dykman and Ackerman, 1991; Kaplan et al., 2006). Children with co-occurring ADHD and dyslexia seem to share common neuropsychological deficits (slower naming speed for letters, impaired phonological processing, and poor word identification or reading), behavioral deficits (impulsivity and inattention), and inhibition deficits (Wolf and Bowers, 1999; American Psychiatric Association, 2000; Donfrancesco et al., 2005; Tiffin-Richards et al., 2008; Laasonen et al., 2012).

Posner and his team proposed in the 1990s that the attention network has three separate main networks associated with attention. These are known as the alerting, orienting, and inhibition networks (Posner and Petersen, 1990; Posner and Raichle, 1994). More recent studies have shown that this theoretical model provides a good fit for examining the potential cognitive mechanisms underlying attentional problems (Berger and Posner, 2000; Mullane et al., 2011) and reading difficulties (Bednarek et al., 2004; Goldfarb and Shaul, 2013). The Attention Network Test (ANT) is a reaction time (RT) task designed to measure these three attention networks in the same task (Fan et al., 2002; Rueda et al., 2004a). In the ANT, the participant must determine the direction of the central arrow or fish (target) surrounded by congruent or incongruent arrows or fish (flankers). The array of arrows or fish is preceded by either an alerting visual cue or a spatially orienting cue. Although the literature on these three attention networks shows some evidence of group differences between controls and individuals with AP (Konrad et al., 2006; Booth et al., 2007; Kratz et al., 2011; Mullane et al., 2011; Fabio and Urso, 2014; Hasler et al., 2016) in terms of reaction time, ERPs, and fMRI (BOLD signal), the results remain somewhat contradictory, as will be described in the following sections.

The alerting sub-processes of attention can be defined as a network associated with arousal and vigilance involved in the attainment and maintenance of a state of sensitivity to subsequent stimuli (Posner and Petersen, 1990). The alerting effect is measured by differentiating stimuli preceded by non-informative visual warning cues and informative cues (Fan et al., 2002). On the other hand, orientation is associated with spatial selection (Neuhaus et al., 2010). Orienting effects can be measured (similar to that for alerting effects) by an RT difference between center-cued and spatially cued target stimuli (Neuhaus et al., 2010). Spatial orientation has three distinct sub-functions: the engagement of visual attention to a particular stimulus, the disengagement of visual attention from a stimulus, and the shifting of visual attention from one stimulus to another (Posner and Petersen, 1990).

Individuals with attentional problems tend to have impairment in the alerting process (Sergeant, 2000; Swaab-Barneveld et al., 2000; Willcutt and Carlson, 2005; Konrad et al., 2006). In line with this, children with the combined subtype (attention deficit and hyperactivity) of ADHD and aged between 7 and 13 years showed a larger alerting effect relative to control children (Booth et al., 2007; Mullane et al., 2011), suggesting that their general level of alertness is lower (Mullane et al., 2011). In contrast, previous ANT studies between ADHD and control children (Adólfsdóttir et al., 2008; Kratz et al., 2011; Fabio and Urso, 2014) and adults (Lundervold et al., 2011) did not show significant group differences in the alerting process but did reveal lower accuracy (as measured by number of correct responses) and higher omission errors in the ADHD group, which indicates a higher level of inattention than vigilance. The orienting effect in 7−13-year-old children (Booth et al., 2007; Adólfsdóttir et al., 2008; Kratz et al., 2011; Fabio and Urso, 2014) and adults (Lundervold et al., 2011) with ADHD did not differ from control groups. The lack of difference between the groups suggests that either the orienting effects might not be affected in children with ADHD, or that the effect was too small to be detected in the previous studies (Huang-Pollock and Nigg, 2003).

The alerting effect observed in the 10-year-old children with dyslexia (Bednarek et al., 2004) and adults with dyslexia (Buchholz and Aimola Davies, 2008) was not significantly different from the control groups, suggesting that both the dyslexia and control groups tend to have an increased level of readiness when a target stimuli is cued (Bednarek et al., 2004; Buchholz and Aimola Davies, 2008). Similarly, dyslexic and control children (10-year-olds) did not show any evidence of a deficit in the orienting effect (Bednarek et al., 2004). In contrast, in the adult studies (Buchholz and Aimola Davies, 2008; Goldfarb and Shaul, 2013) there was a significant group difference in the orienting effect between the dyslexic and control groups. A study with dyslexic adults showed that such individuals have difficulties in the adjustment and maintenance of attentional focus and peripheral spatial location (Buchholz and Aimola Davies, 2008).

The functioning of the attentional processes at the brain level has been widely investigated using the ANT paradigm coupled with ERPs in children (Kratz et al., 2011; Santhana Gopalan et al., 2019) and adults (Neuhaus et al., 2010; Kratz et al., 2011; Rueda et al., 2012; Kaufman et al., 2016). Generally, alerting (non-cued vs. visually cued target stimuli) and orienting visual cues (center-cued vs. spatially cued target stimuli) enhance the modulation of the posterior visual N1 amplitude at 100–280 ms for the target stimulus (Hillyard and Anllo-Vento, 1998; Neuhaus et al., 2010; Luck, 2014; Kaufman et al., 2016). In children and adults (Fan et al., 2005; Neuhaus et al., 2010; Galvao-Carmona et al., 2014), target stimulus-related N1 was modulated by cue conditions (double, spatial, and center) over the occipital and parietal regions, reflecting the visual attentional processing of target stimulus properties in relation to the cue context. Studies in adults have consistently shown that spatially cued target stimuli elicit a larger N1 amplitude than center-cued target stimuli, which suggests stronger engagement and lasting effects for the spatial cue with regard to the target stimulus (Kaufman et al., 2016; Williams et al., 2016). Alerting and orienting N1 amplitudes in adults with ADHD follow a similar pattern as that for control adults, which corroborates the reaction time studies on adults with ADHD (López et al., 2006; Hasler et al., 2016). However, there are no studies showing how N1-alerting and orienting effects for a visually cued target stimulus are processed in AP and RD children.

Functional magnetic resonance imaging studies have revealed that several brain sources are activated during the attention network test. The alerting network in adult fMRI studies has been shown to have increased neuronal activity in the thalamus, temporal parietal junction (TPJ), and prefrontal cortex (Fan et al., 2005; Konrad et al., 2005). A recent adult fMRI study produced results with additional brain areas in the anterior cingulate cortex (ACC), frontal eye fields (FEF), occipital, and visual areas (Xuan et al., 2016). The alerting network in an fMRI study of children showed increased neuronal activity in the bilateral occipital lobe and temporal lobe (i.e., the middle occipital cortex extending toward the right superior temporal gyrus), suggesting that these regions enhance the anticipation of the visual warning cue and response preparation toward the upcoming target stimuli (Konrad et al., 2005; Xuan et al., 2016). There is some evidence from fMRI studies that the alerting network might activate differently in children with ADHD. In control children, the right ACC showed greater activation compared to ADHD children, suggesting that neural activity is modulated with a top-down bias in control children, thereby assisting in the processing of stimuli at the attended location (Sturm and Willmes, 2001; Konrad et al., 2006).

The orienting network in fMRI studies with adults has shown neuronal activity in the TPJ, bilateral superior parietal lobe, FEFs, pulvinar, and superior colliculus (Fan et al., 2005; Konrad et al., 2005; Xuan et al., 2016). Previous ANT studies with children found orienting network responses in the superior frontal gyrus and bilaterally in the occipital cortex (Konrad et al., 2005; Santhana Gopalan et al., 2019). A previous fMRI study also showed that children with ADHD have atypical activation in the frontostriatal region compared to control children (Bellman, 2002, 104). This altered brain activation could be due to an alternative function (brain functions to solve a problem and not necessarily to an overt or volitional approach used by the children) during reorienting, which includes the dorsolateral prefrontal cortex and insular cortex (Bellman, 2002, 104; Konrad et al., 2006). No studies have investigated the neural sources associated with the alerting or orienting networks in individuals with RD using fMRI.

The third attention network tapped by the ANT is related to inhibition. Inhibition involves a number of mechanisms for resolving conflicts, detecting errors, and selecting actions in response to target stimuli (Michael, 1998; Posner and Rothbart, 2007). The inhibition effect in the ANT is measured by the RT difference between incongruent and congruent target stimuli (Fan et al., 2002; Neuhaus et al., 2010).

Several studies with children (involving 7−13-year-olds) (Booth et al., 2007; Adólfsdóttir et al., 2008; Kratz et al., 2011) and adults (Lundervold et al., 2011) have shown that ADHD children and control groups do not differ with respect to the inhibition effect in ANT. However, one study with ADHD children showed larger inhibition effects (i.e., more time to change the focus when the stimulus is incongruent) relative to a control group (Fabio and Urso, 2014). According to the authors, this indicates that ADHD children could have a deficit in inhibition processes (Fabio and Urso, 2014). The inhibition effect in the 10-year-old dyslexic children (Bednarek et al., 2004) and adults (Goldfarb and Shaul, 2013) showed slower reaction time performance compared to their respective control groups. These findings were interpreted as representing an executive control deficiency in the inhibition of distracting information (Bednarek et al., 2004; Goldfarb and Shaul, 2013).

At the brain response level in ERP studies, inhibition effects have been associated with a P3 response in the time window between 300–650 ms from target stimulus onset (Neuhaus et al., 2010; Kratz et al., 2011; Mahé et al., 2014; Hasler et al., 2016; Kaufman et al., 2016). In the context of ANT, the P3 response represents the neural activity related to the processing of cueing information for target detection (Neuhaus et al., 2010) and response control processes to target stimuli (motor selection and inhibition) (Polich, 2007). In ANT, a target stimulus-generated P3 response is generally observed with delayed latency in children (4–12 years old) (Rueda et al., 2004b; Kratz et al., 2011) compared to adults (Neuhaus et al., 2010; Kaufman et al., 2016), which suggests a developmental trend in the evaluation of the target direction (Falkenstein et al., 1994). Furthermore, previous studies have shown that the target P3 in ANT has smaller amplitudes in predominantly inattentive 10-year-old children (Kratz et al., 2011), ADHD adults (Hasler et al., 2016), and dyslexic adults (Mahé et al., 2014) compared to control groups, which suggests an impairment in attentional resource allocation leading to decreased target stimulus evaluation and processing capabilities for a difficult task (Mahé et al., 2014; Hasler et al., 2016). No studies have used the children’s version of the ANT to examine inhibition effects in children with RD.

Attention Network Test studies on adults using fMRI have revealed activation related to inhibition in the right ACC, bilateral precentral gyrus, intraparietal sulcus, anterior insular cortex, FEFs, and bilateral occipital cortex (Xuan et al., 2016). In children eight to 12 years old, inhibition processes (ANT experiment) activated the right superior temporal gyrus, bilateral parietal, occipital, and premotor cortices but involved less prefrontal cortex activation (inferior and medial frontal gyrus) compared to adults (Bunge et al., 2002; Konrad et al., 2005). In line with the reaction time and ERP studies, fMRI studies also show differences between children with ADHD and control children with respect to inhibition networks. Specifically, ADHD children (8−12 years old) showed typical left superior parietal cortex and posterior parietal cortex activations (Durston et al., 2003; Konrad et al., 2006) and reduced frontostriatal activation compared to control children. Together, the results provide strong evidence that in children with ADHD, there is decreased activation or immature frontal development of the inhibition network (Bunge et al., 2002; Durston et al., 2002, 2003; Konrad et al., 2006). With respect to the examination of regions associated with the inhibition network, there have been no fMRI studies of children with RD that have assessed attentional processing using the visual attentional task.

In summary, Posner’s model of attention (Posner and Raichle, 1994) and previous neuroimaging studies show that the alerting network involves the thalamus, TPJ, prefrontal cortex, occipital and visual areas associated with readiness, arousal, and vigilance (Fan et al., 2005; Konrad et al., 2005; Booth et al., 2007). Neuroimaging studies of control groups have consistently shown the occipital cortex and TPJ to subserve the orienting of attention (Corbetta and Shulman, 2002). Neurologically, the right TPJ receives information from various brain areas about stimuli in the environment and inhibits spatial orientation (Goldfarb and Shaul, 2013). Developmental changes in the right TPJ have been linked to reading acquisition in normally developing children, and some studies have observed differences in the activation of the right TPJ in dyslexics (Grünling et al., 2004; Hoeft et al., 2006). The inhibition network involves the prefrontal cortex (including ACC and FEFs) and the parietal cortex associated with conflict resolution appears to be deficient in individuals with ADHD (Konrad et al., 2006; Booth et al., 2007). These findings provide a basis for examining the three attentional networks in AP and RD groups.

Some studies using ANT have utilized EEG (Neuhaus et al., 2010; Kratz et al., 2011; Williams et al., 2016) and fMRI (Fan et al., 2005; Konrad et al., 2005; Rueda et al., 2012; Xuan et al., 2016) to demonstrate the time course and network of attention-related brain activations. However, EEG-based studies of attentional sub-processes in school-aged children with AP and RD groups are rare (Kratz et al., 2011). Although reaction time performance in AP and RD children and the target stimulus P3 in AP children have been examined, there remains a lack of knowledge about the target stimulus N1 in AP and RD children as it relates to alerting (double-cued vs. non-cued target stimuli) and orienting (spatially cued vs. center-cued target stimuli) processes. This is also the case for target stimulus P3 in RD children (incongruent vs. congruent target stimuli). Further investigation of neuronal sources in AP and RD children that capitalizes on high temporal resolution EEG by using source models based on typically developing children to identify the brain areas associated with these three attentional networks would address this knowledge gap. This would help us to understand the time course of activation in the different brain regions involved in the attention network of children with RD or AP.

In this study, we investigated reaction time performance during the ANT (as modified for children) and the modulation of the target-stimulus-driven N1 amplitude related both to the alerting and orienting networks, the modulation of the P3 amplitude related to the inhibition network, and their neural sources in children with attentional and reading difficulties. We employed source models derived from the data of control children (Santhana Gopalan et al., 2019) as a spatial filter for the source localization of the three network effects in AP and RD children.

Based on previous ANT reaction time studies, we could assume that the alerting and inhibition effects in AP children (Konrad et al., 2006; Booth et al., 2007; Fabio and Urso, 2014) and the inhibition effect in RD children (Bednarek et al., 2004), would be different compared to a control group. Previous studies have not found such group effects (Adólfsdóttir et al., 2008; Kratz et al., 2011; Lundervold et al., 2011) and have emphasized the importance of replication. We expected that differences in inhibition effect in AP children would produce reduced P3 amplitude associated with target-related attentional processes. This could reflect the atypical function of the TPJ and ventral frontal cortex involved in the processing of the stimulus (Szuromi et al., 2011; Vossel et al., 2014; Hasler et al., 2016). Furthermore, in line with previous fMRI studies (Fan et al., 2005; Konrad et al., 2005; Xuan et al., 2016) and our previous EEG investigation (Santhana Gopalan et al., 2019), we assumed that alerting effects in children with AP would modulate atypical activity in the bilateral occipital lobe and temporal lobe compared to control children. Orienting would modulate atypical activity in the bilateral occipital lobe compared to controls, and inhibition would modulate atypical activity in the bilateral occipital lobe, parietal lobe, and prefrontal cortices as compared to a control group. We did not hypothesize brain regions for any of the three attention sub-networks in children with RD because we did not find any literature on this issue.

## Materials and Methods

### Participants

**FIGURE 1:**
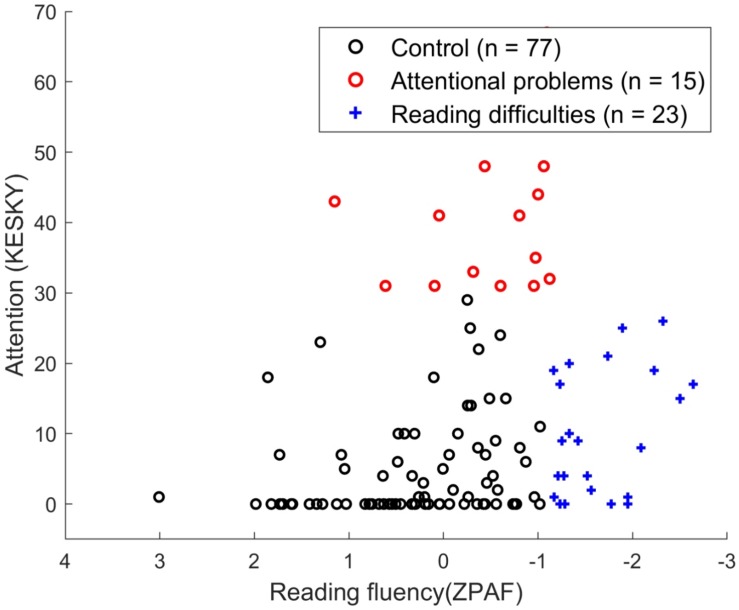
Distribution of control (*N* = 77; 36 boys, 41 girls), attentional problems (*N* = 15; 14 boys, 1 girl), and reading difficulties (*N* = 23; 15 boys, 8 girls) groups based on the reading fluency score (as evaluated using a composite score derived from the word identification test, the word chain test, and the oral pseudoword text reading test) and attention score (ATTEX). Symbols for each group are as follows: control (black circle), AP (red circle), and RD (blue cross).

The data consist of 115 (65 boys, 50 girls) Finnish sixth-graders with normal visuospatial reasoning ability aged between 12 and 13 years. Inclusion criteria for the attentional problems (AP) group (*N* = 15; 14 boys, 1 girl) (mean age: 12.67 years, SD: 0.31) were as follows: an ATTEX score above 30 (Klenberg et al., 2010) and a reading fluency score above the 10th percentile [which is a composite score of three reading tasks created using Principal Axis Factoring, PAF (see detailed description below)]. For the reading difficulties (RD) group (*N* = 23; 15 boys, 8 girls) (mean age: 12.61 years, SD: 0.31), an ATTEX score below 30 and a reading fluency score below the 10th percentile were the criteria for inclusion. Inclusion criteria for the control group (*N* = 77; 36 boys, 41 girls) (mean age: 12.86 years, SD: 0.31) were an ATTEX score below 30 and a reading fluency score above the 10th percentile (see Figure 1). The control sample used in this study was the same as in our previous study (Santhana Gopalan et al., 2019) with the exclusion of six participants because they were below the borderline in their reading skills to be included in the control group based on the updated criteria for the reading disorder in the current study. This exclusion did not alter any of the results. Children with both AP and RD were excluded in this study because of sample size (*n* < 10) (these children had an ATTEX score above 30 and a reading fluency score below the 10th percentile). All children had normal or corrected vision and no history of neurological problems or head injuries, which was reported by parents or guardians. The study was conducted in compliance with the Declaration of Helsinki, and protocols were approved by the ethics committee of the University of Jyväskylä, Finland. All methods were performed in accordance with relevant guidelines and regulations. The participants and their parents signed a letter of informed consent prior to the experiment.

In this study, 466 participants were recruited from sixth grade schools in Central Finland during the years 2014–2015. Schools from both rural and urban areas participated voluntarily in this study. Participants (numbering 448) finished the ILA test (Kiili et al., 2018a, b; Kanniainen et al., 2019). The test comprises simulated closed internet environmental tasks that measure an individual’s ability to: (1) locate information, (2) evaluate information, (3) synthesize information, and (4) communicate information (Leu et al., 2013; Kiili et al., 2018b). One hundred and fifty-six participants were invited to the EEG measurement based on the completion of the ILA test and performance in the RAVEN test (Raven and Court, 1998). The AP and RD participants were included based on ATTEX and reading fluency (PAF) scores. Detailed selection criteria are described below. The participants who did not complete the ILA test and whose shortened RAVEN score was less than 15 were not invited to the individual EEG measurements. Participants with a native language other than Finnish were not included in this study.

### Behavioral Measures

Reading fluency, attention, and visuospatial reasoning ability of the children were assessed during the sixth grade (see Table 1).

**TABLE 1:** Summary of reading fluency, attention, and executive function rating inventory (ATTEX), Raven’s Standard Progressive Matrices test, block design test, and their statistics between groups.

Group		Reading fluency				ATTEX				RAVEN				Block design			
					
	df	*t*-value	*p*-value		Cohen’s d	*t*-value	*p*-value		Cohen’s d	*t*-value	*p*-value		Cohen’s d	*t*-value	*p*-value		Cohen’s d
**Control vs. AP**	90	2.914	0.004		0.822	−16.342	0.000		−4.612	1.589	0.115		0.448	3.454	0.001		0.975
**Control vs. RD**	98	10.451	0.000		2.483	−2.906	0.005		−0.691	1.953	0.054		0.464	1.270	0.207		0.302
**AP vs. RD**	36	6.567	0.000		2.179	9.651	0.000		3.203	−0.023	0.982		−0.008	−1.496	0.143		−0.496

		***M***		***SD***		***M***		***SD***		***M***		***SD***		***M***		***SD***	

**Control**		0.283		0.851		4.82		7.150		23.34		3.059		46.60		8.367	
**AP**		0.401		0.719		40.80		10.665		21.93		3.494		38.13		10.225	
**RD**		1.654		0.461		10.04		8.860		21.96		2.671		43.78		12.060	

*The *t*-values denote test statistics with degrees of freedom (df). AP denotes the attentional problems group, RD denotes the reading difficulties group, and control denotes the typically developing group. Cohen’s d denotes the effect size between groups. *M* and *SD* denotes the mean and standard deviation of each test score in the three groups. The FDR corrected alpha value is 0.012.*

Reading fluency performance was evaluated using a composite score derived from the following three subtests using PAF with Promax rotation. The factor analysis was forced into one factor. The word identification test and word chain test were conducted as a group session. The oral pseudoword text-reading test was conducted as an individual session.

(1) The word identification test, which is a subtest of the standardized Finnish reading test ALLU (Lindeman, 1998), consists of 80 items, each consisting of a picture and four phonologically similar words, one of them semantically matching the picture. The purpose of the task was to identify and connect correct picture-word pairs as quickly as possible by drawing a line between a word and the picture. The maximum duration of the task was 2 min. The score was the number of correctly connected pairs completed within the time limit. The Kuder-Richardson reliability coefficient for the original test is 0.97 (Lindeman, 1998). The factor loading of the test for the reading fluency factor is 0.683.
(2) The word chain test (Holopainen et al., 2004) consists of 25 chains, each consisting of four words written without spaces between them. The task was to insert a vertical line at the word boundaries. The maximum duration was 90 s, and the score was the number of correctly separated words within the time limit. The test-retest reliability coefficient for the original test varied between 0.70 and 0.84. The factor loading of the test for the reading fluency factor is 0.872.
(3) The oral pseudoword text-reading test (Eklund et al., 2015) consists of 38 pseudowords (277 letters). These pseudowords were given as a short passage, which children were instructed to read aloud as quickly and accurately as possible. The reading performance of the students was audio recorded for scoring. The score was the number of correctly read pseudowords divided by the time (in seconds) spent on reading. The inter-rater agreement for scoring the original test is 0.95. The factor loading of the test for the reading fluency factor is 0.653.

Attention and executive function rating inventory (ATTEX) (Klenberg et al., 2010) is a teacher rating scale with 55 items to measure difficulties of inhibition, attention, and executive function in school settings grouped into ten clinical subscales (number of items per scale in parentheses): distractibility (4), impulsivity (9), motor hyperactivity (7), directing attention (5), sustaining attention (6), shifting attention (4), initiative (5), planning (4), execution of action (8), and evaluation (3). The teachers were instructed to rate the child’s behavior on a three-point scale (“not a problem,” “sometimes a problem,” and “often a problem”). The internal consistency reliability of ATTEX and its scales varies between 0.67–0.98 and criterion validity varies between 0.68–0.95 (Klenberg et al., 2010).

Visuospatial reasoning ability was evaluated based on the following two subtests:

(1) Non-verbal reasoning ability was assessed using the Raven’s Standard Progressive Matrices (RSPM) test, which is a visuospatial task (Raven and Court, 1998; John and Raven, 2003). This was conducted as a group testing session. The test consists of 60 items, of which a shortened version was used containing 30 items (every second item from the complete test). The task was to select the one correct option among six to eight choices to fill in a missing part and complete a picture matrix. These choices were always similar in shape, but they varied from each other with respect to their pattern. The total score was the number of items correctly responded to. The maximum duration of the task was 15 min. In another large-scale project with more than 800 sixth graders from the same area in Finland, the same shortened version was used with a Cronbach’s alpha reliability coefficient of 0.81 (Kanerva et al., 2019).
(2) A block design test (WISC-IV) (Lynne Beal, 2004) was used to measure spatial ability. It consists of nine red and white square blocks and a booklet of cards with different color designs that can be made with the blocks. The task was to arrange the blocks to match the design formed by the examiner (or as shown on cards) as quickly and accurately as possible. This test was used to further characterize the groups and was not used as an inclusion or exclusion criterion.

### Experimental Procedure: Attention Network Test for Children

In this EEG experiment, a modified version of the ANT (Neuhaus et al., 2010) was used to measure the three sub-processes of the attention network: alerting, orienting, and inhibition. Participants were required to lean on a chinrest located 60 cm from a 24-inch computer screen (resolution of 1920 × 1080 and a refresh rate of 60 Hz). A fixation cross was visible in the center of the white screen [960, 540 (x,y)] during the entire testing period. The participant’s task was to look at the fixation cross and report the direction of the middle fish as quickly and accurately as possible by pressing a corresponding button.

**FIGURE 2:**
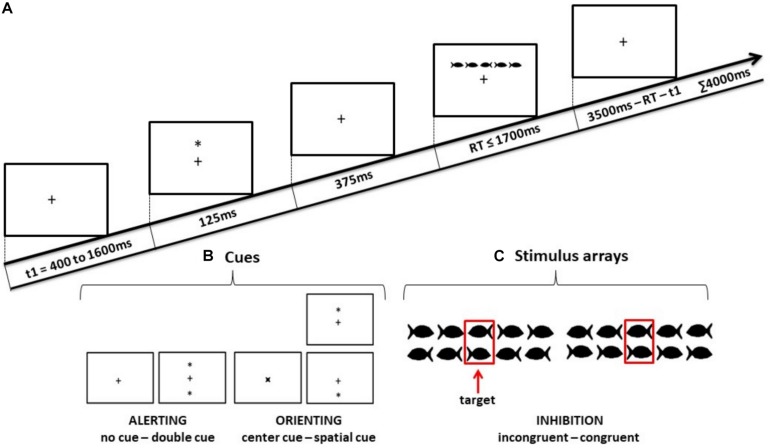
Schematic illustrations of **(A)** the sequence of events in the Child-Attention Network Test (ANT), **(B)** the four cue conditions used in ANT, and **(C)** the two target stimulus conditions for which the children had to decide the swimming direction of the middle fish.

As shown in Figure 2, the stimulus (a group of fish) was preceded by one of the four cue conditions (no cue, double cue, center cue, or spatial cue). The fixation period of a random duration was between 400 ms and 1600 ms before the cue appeared. The duration of the cue was 125 ms, which was followed by 375 ms of waiting time before the stimulus was presented (a total of 500 ms prior to stimulus presentation). In the double cue trial, two asterisks were presented simultaneously at a 1° angle above and below the fixation cross. In the center cue trial, an asterisk was presented on the fixation cross. In the spatial cue trial, a single asterisk appeared in the position of the upcoming stimulus.

To make the experiment more child-friendly, black fish drawings instead of arrows were used as stimuli. The stimulus comprised a row of five horizontal fish. Each fish was subtended to 0.7°, and adjacent fish were separated by 0.3° each. The size of the entire stimulus array was 4.7°. The center fish in the stimulus was the target, and the two fish on either side of the target were referred to as flankers. The stimulus array in each trial was presented above or below the fixation cross at the same location where the double cue or spatial cue appeared. The maximum duration of each trial was 4000 ms. The maximum duration of the stimulus array in each trial was 1700 ms until a response was detected; thereafter, if there was no response, it was considered an unattended trial and terminated. The maximum duration between the onset of the stimulus and the start time of the next trial was 3500 ms, which varied according to the duration of the stimulus array. For congruent stimuli, the flankers were in the same direction as the target and for incongruent stimuli, the flankers were in the opposite direction. Participants were instructed to keep their gaze on the fixation cross throughout the experiment and report the swimming direction of the center fish by pressing a left or right corresponding direction button in the button box.

One ANT session consisted of 288 pseudo-randomized trials, which were divided into four experimental blocks with 72 trials in each block. Each block consisted of all eight possible conditions in equal proportions: four cue conditions (no cue, double cue, center cue, and spatial cue) × two target stimulus conditions (congruent, incongruent).

### EEG and Eye-Tracker Recording

**FIGURE 3:**
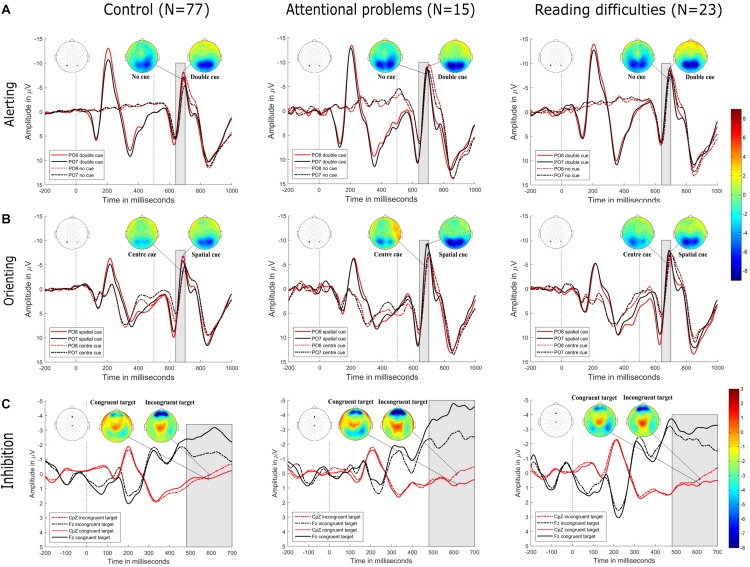
**(A)** Alerting. Grand-averaged ERP waveforms for the double-cued target stimulus (solid lines) and non-cued target stimulus (dotted lines) at posterior electrodes (PO8, red, right hemisphere; PO7, black, left hemisphere) in control (top row-left), attentional problems (top row-center), and reading difficulties (top row-right) groups. Cue onset is at 0 ms and target stimulus onset is at 500 ms. Amplitude topographies for double-cued and non-cued target stimuli are at 689 ms (i.e., 189 ms after target stimulus onset). **(B)** Orienting. Grand-averaged ERP waveforms for the spatially cued target stimulus (solid lines) and center-cued target stimulus (dotted lines) for posterior electrodes (PO8, red, right hemisphere; PO7, black, left hemisphere) in control (middle row-left), attentional problems (middle row-center), and reading difficulties (middle row-right) groups. Cue onset is at 0 ms and target stimulus onset is at 500 ms. Amplitude topographies for spatially cued and center-cued target stimuli are at 686 ms (i.e., 186 ms after target stimulus onset). **(C)** Inhibition. Grand-averaged ERP waveforms for the congruent stimulus (dotted lines) and incongruent stimulus (solid lines) at central electrode (CpZ, red) and frontal electrode (Fz, black) in control (bottom row-left), attentional problems (bottom row-middle), and reading difficulties (bottom row-right) groups. Target stimulus onset is at 0 ms. Amplitude topographies for congruent and incongruent target stimuli are at 612 ms after target stimulus onset. Negativity is upward.

The ANT experiment was designed using the Experiment Builder (1.10.1630) software on a Dell Precision T5500 workstation. Electroencephalography data were recorded using a high-density array of 128 Ag-AgCl electrodes in HydroCel Geodesic Sensor Nets (GSN; Electrical Geodesics Inc.). The electrode positions for 128 channel HydroCel GSN approximate the correspondence with the international 10–10 system electrode positions. The electrode numbers 11, 55, 65, and 90 plotted in Figure 3 correspond to Fz, CpZ, PO7, and PO8, respectively, based on the international 10–10 system (Luu and Ferree, 2000). The EEG data were amplified using a NeurOne amplifier (Mega Electronics Ltd.). During measurement, the impedance of the electrodes was intended to be kept below 50 kΩ, and the quality of the EEG data was monitored throughout the EEG recording. Electroencephalography was referenced to the Cz electrode online and sampled at 1000 Hz. An online high-pass filter of 0.16 Hz and a low-pass filter of 250 Hz were applied during EEG data recording. Eye movement data were recorded with a table-mounted Eyelink 1000 eye-tracking device at 1000 Hz for both eyes (SR Research Ltd.). Eye movements and EEG were recorded simultaneously through the combination of triggering via ethernet messages and TTL pulses. The entire experiment was conducted in a dimly lit sound-attenuated room in a laboratory at the University of Jyväskylä, Finland.

### Pre-processing of EEG Data and Eye Tracking

Electroencephalography data were preprocessed using MatLab R2014a, with EEGLab (Delorme and Makeig, 2004) (Swartz Centre for Computational Neuroscience, San Diego), FieldTrip (Oostenveld et al., 2011) (version 20160110) toolboxes, and BESA Research 6.1 (BESA GmbH, Munich, Germany). The raw eye tracking data were converted and stored into a MatLab structured array using the EEGLab add-on EYE-EEG (Dimigen et al., 2011). The continuous raw EEG data file was imported into EEGLab, in which bad channels were interpolated. In EEGLab, the events observed in EEG and eye tracking were used for synchronization. One event at each of the beginning and end of the eye-tracking data record were linearly interpolated to match the number of EEG sampling points recorded during the same time interval. The quality of synchronization was assessed by examination of the linear regression line for the regression of latencies of eye-tracking events on the latencies of EEG events.

A high-pass filter of 0.5 Hz (a fifth order, zero-phase Butterworth filter) was applied to the raw EEG data. Data were segmented into 1200 ms epochs (200 ms before cue onset and 1000 ms after cue onset) for non-cued, double-cued, center-cued, and spatially cued target stimuli, and 900 ms epochs (200 ms before and 700 ms after the onset of target stimulus) for congruent and incongruent stimuli. Trials with incorrect responses were excluded from the data analyses. A low-pass filter of 30 Hz (sixth order, zero-phase Butterworth filter) was then applied to the high-pass filtered, segmented EEG data. The baseline was set to −200 ms and 0 ms of the filtered segmented data. Gaze positions in each trial were examined in order to ensure that participants maintained their gaze in the optimal position for stimulus presentation. If there was an eye blink, the gaze position value was recorded as zero, or if the gaze position was outside the defined area [860–1060, 440–640 (x,y)] on the display screen, the trial was excluded. Trials with muscular movement and other artifacts were rejected using a threshold rejection approach. The value of threshold rejection was 175 μV. The average percentages of rejected trials for all conditions in control, AP, and RD groups are given in Supplementary Table 1. Accepted trials using the above criteria were averaged for each participant. The averaged ERPs were re-referenced to the average reference. In the control group, each condition (non-cued, double-cued, center-cued, spatially cued, congruent, and incongruent target stimuli) had a minimum of 30 trials for averaging. In the attentional problems and reading difficulties groups, one subject had a minimum of 22 trials in the non-cued and cued stimulus conditions for averaging, while two participants had a minimum of 24 trials in a no-cue condition for averaging. The remainder had a minimum of 30 trials. The averaged data were visually inspected and comparable to that of other participants.

### Statistical Analysis of Reaction Time Data

The RTs of each trial were calculated from the target stimulus onset time to the button press response time. The unattended trials, trials with incorrect responses, and trials that were not accepted for ERP averaging were excluded from calculations of the mean RTs. All participants maintained a high level of accuracy (see Supplementary Table 3). There were no participants excluded due to poor performance. Repeated measures ANOVAs were performed in IBM SPSS Statistics version 24 to determine significant differences in RTs between conditions and groups. Separate Repeated measures ANOVAs for alerting, orienting, and inhibition {3 (group) × 2 (condition)} were calculated with repeated measures to determine the significance of the reaction time performance between the groups (control, AP, RD) and conditions (alerting: non-cued and double-cued target stimuli; orienting: center-cue and spatially cued target stimuli; inhibition: incongruent and congruent target stimuli). Paired-sample t-tests were calculated in IBM SPSS Statistics version 24 to determine the significant differences in RTs between conditions within the groups. Cohen’s D_z_ was calculated to determine the effect size between RTs for different target stimuli within a group.

### Statistical Analysis of ERP Responses at the Sensor Level of Field Potentials

Non-parametric, cluster-based permutation tests were calculated as a two-tailed test using BESA Statistics 2.0 (BESA GmbH, Munich, Germany) to determine significant effects for the field ERP field potentials across all the electrodes between conditions (alerting: double-cued vs. non-cued target stimuli; orienting: spatially cued vs. center-cued target stimuli; inhibition: incongruent vs. congruent target stimuli) within the groups. The difference waveform was calculated between the conditions (alerting: double-cued vs. non-cued target stimuli; orienting: spatially cued vs. center-cued target stimuli; inhibition: incongruent vs. congruent target stimuli) using BESA Research 6.1. Non-parametric, cluster-based permutation tests were then calculated as a two-tailed test to determine the significant effects for the difference in wave ERP field potentials across all the electrodes between groups (control vs. AP; control vs. RD; AP vs. RD). Based on our previous study (Santhana Gopalan et al., 2019), the time window for cluster-based permutation tests between groups was set to 140–200 ms after target onset (alerting and orienting conditions) and 480–700 ms after target onset (inhibition conditions). The number of permutations was set to 1000, and cluster alpha (the significance threshold level for data to enter a cluster) was set to 0.05. For spatial clustering, the neighbor distance between electrodes was set to 3 cm.

### Source-Level Analysis

Source analysis was performed in BESA Research 6.1 to estimate source areas in the brain related to the sub-processes of attention. In our previous study (Santhana Gopalan et al., 2019), we reconstructed the source representation of scalp data based on the control children (*N* = 83) using the classical LORETA analysis recursively applied (CLARA) distributed source analysis method. A regional source was fitted in the foci obtained from the CLARA solution. A regional source was considered as three single dipoles at the same location, with three orthogonal orientations (Hoechstetter et al., 2010). The source strength at each time point was estimated as a combined sum of the power of the three orthogonal orientations of the regional sources. These regional sources were used as a spatial filter for source modeling for each of the three effects in the control children. The spatial filter with regional sources derived from the control group data was used to obtain the strength of the source activity for each stimulus condition in AP and RD groups, i.e., the scalp data of AP and RD groups were “projected” into the sources derived using the control group data. The time window of interest for the N1 period of the target stimulus was between 140 and 200 ms, and the time window for the P3 period of the target stimulus was between 480 and 700 ms.

### Source-Level Analysis Statistics

Residual variance was examined in BESA Research 6.1 to determine the goodness of fit of the regional source model for the neuronal data in each condition and each group (see Supplementary Table 2). T-tests for the residual variance were calculated using SPSS version 24 to confirm that there was no difference between conditions and groups. There was no significant group difference between any groups (control vs. AP; control vs. RD; AP vs. RD) with respect to the residual variance. The activity in each source was initially compared against zero using a *t*-test to determine if a signal was present in the source. The source activity of the left anterior temporal lobe in the alerting and orienting networks did not show a significant difference from zero. This source was therefore excluded from further analysis. Source-level statistics were calculated using a 2 (conditions) × 3 (groups) repeated measures ANOVAs in SPSS version 24. Statistical analyses considered cued-target conditions and congruency target conditions as within-subjects factors. Between-subjects factors included the control, AP, and RD groups. For the source level statistics, N1 (140–200 ms) and P3 (480–700 ms) cued-target stimulus periods were selected from the source waveforms associated with the locations of the neuronal sources. The repeated measures ANOVA with trials as covariates, and group and condition as factors for neuronal source were checked to confirm that the number of trials did not affect the interaction between the groups. To correct for the multiple comparisons regarding RT and neuronal sources, we adjusted the alpha level using the false discovery rate method with *q* = 0.05 (Benjamini and Hochberg, 1995; Benjamini and Yekutieli, 2001, 2005). After correction, the *p*-Values smaller than or equal to the corrected alpha value (0.0120) were considered significant.

## Results

### Behavioral Tests

Reaction time performance on the ANT was first examined to verify the existence of alerting, orienting, and inhibition effects, as well as possible differences between the groups. The repeated measures ANOVA (see Table 2) indicated significant main effects for the condition for alerting (non-cued vs. double-cued target stimuli), orienting (center-cued vs. spatially cued target stimuli), and inhibition (incongruent vs. congruent target stimuli) sub-processes and main effects of group with respect to RT performance. For alerting, the main effect of condition indicated a decrease in RT on double-cued target stimuli relative to non-cued target stimuli. For orienting, the main effect of condition indicated a decrease in RT on spatially cued target stimuli relative to center-cued target stimuli. For inhibition, the main effect of condition indicated an increase in RT on an incongruent target relative to a congruent target. No significant interactions between conditions and groups were found. The main effect of group was significant across the alerting, orienting, and inhibition conditions, indicating that the overall reaction time in children with RD was longer than that for control children and children with AP.

**TABLE 2:** Repeated measures ANOVA test statistics for reaction time performances between controls (*N* = 77), children with attentional problems (*N* = 15), and children with reading difficulties (*N* = 23).

		Alerting (no cue vs. double cue)			Orienting (center cue vs. spatial cue)			Inhibition (incongruent vs. congruent)		
				
	df	*F*	*P*	η^2^_p_	*F*	*P*	η^2^_p_	*F*	*P*	η^2^_p_
Main effect of condition	1	189.995	0.000	0.625	80.097	0.000	0.417	520.692	0.000	0.823
Condition × group interaction	2	1.603	0.206	0.028	3.291	0.041	0.056	2.244	0.111	0.039
Main effect of group	118	5.720	0.004	0.093	6.529	0.002	0.104	6.738	0.002	0.107

*df, denotes degree of freedom; *η*^2^_p_, partial eta-squared. The FDR corrected alpha value is 0.012.*

Within-group *post hoc* t-tests on RT performance (Supplementary Table 3) showed significant differences between all conditions (Alerting: non-cued vs. double-cued target stimuli, Orienting: center-cued vs. spatially cued target stimuli, Inhibition: incongruent vs. congruent target stimuli). Between-group *post hoc* t-tests on RT performance for each condition showed no significant differences after the alpha value correction (Supplementary Table 4).

### Event-Related Field Potentials

Figure 3 shows the grand-averaged ERPs of control, AP, and RD children at electrodes located at bilateral occipital and fronto-central sites. From the onset of the target stimulus, related N1 (140–200 ms) and P3 (480–700 ms) waveforms for these three groups showed similar patterns without observable significant differences between groups. On the other hand, there was a significant difference between conditions within each group for the alerting and orienting effect in the time window from 140 ms to 200 ms. the inhibition effect showed a significant difference between conditions within each group in the time window from 480 to 700 ms.

### Neuronal Sources of ERPs

**FIGURE 4:**
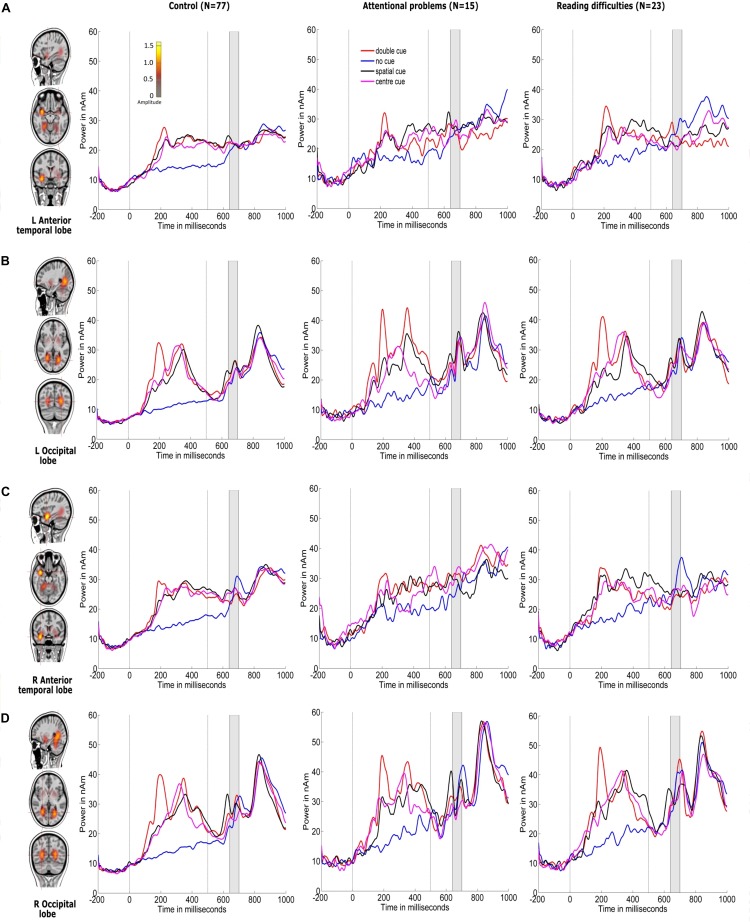
Source locations of grand-averaged ERPs collapsed across all conditions (congruent and incongruent target stimuli, including non-cued, double-cued, center-cued, and spatially cued target stimuli) over time points of the N1 period of the target stimulus (140–200 ms) using CLARA in control children. CLARA was used as a model to derive the source waveforms for control children (*N* = 77), children with attentional problems (*N* = 15), and children with reading difficulties (*N* = 23). Grand-averaged source waveforms were extracted for double-cued (red), non-cued (blue), spatially cued (black), and center-cued (magenta) target stimuli using regional sources at the foci revealed by CLARA (shown on the right side of each source). Cue onset is at 0 ms and target stimulus onset is at 500 ms. Brain activations were localized in the **(A)** left anterior temporal lobe, **(B)** left occipital lobe, **(C)** right anterior temporal lobe, and **(D)** right occipital lobe. The color bar denotes source amplitude. The shaded gray area denotes the source analysis time window.

**FIGURE 5:**
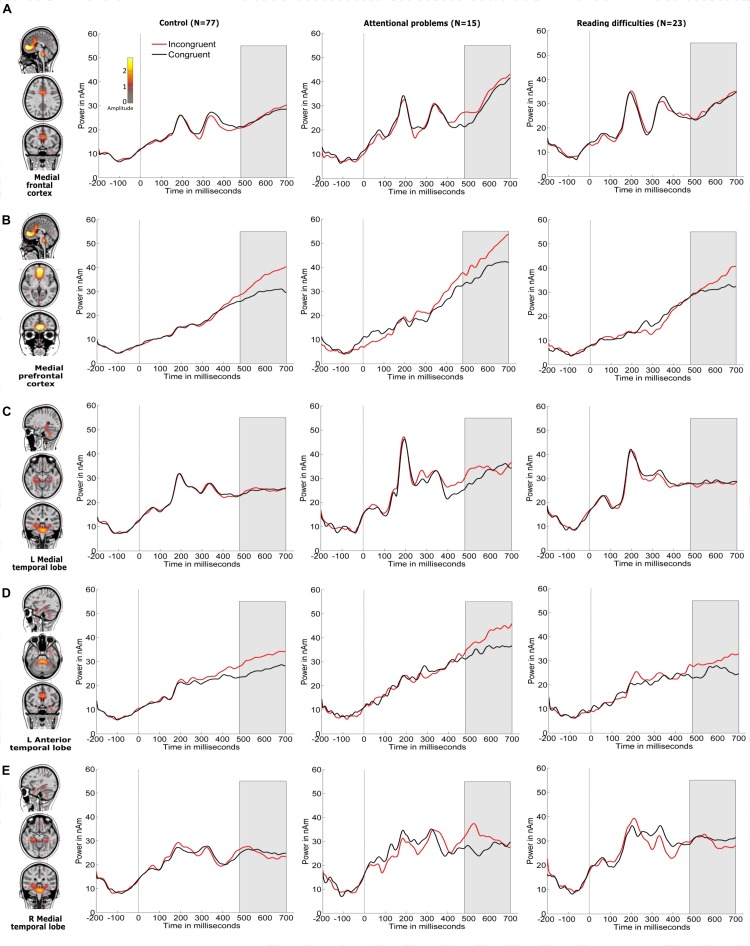
Source locations of grand-averaged ERPs collapsed across all conditions (congruent and incongruent target stimuli, which include non-cued, double-cued, center-cued, and spatially cued target stimuli) over time points of the P3 period of the target stimulus (480–700 ms) using CLARA in control children. CLARA was used as a model to derive the source waveforms for control children (*N* = 77), children with attentional problems (*N* = 15), and children with reading difficulties (*N* = 23). Grand-averaged source waveforms were calculated for incongruent (red) and congruent (black) target stimuli and extracted using regional sources at the foci, as revealed by CLARA (these are shown on the right side of each source). Target stimulus onset is at 0 ms. Brain activations were localized in the **(A)** medial frontal cortex, **(B)** medial prefrontal cortex, **(C)** left medial temporal lobe, **(D)** left anterior temporal lobe, and **(E)** right medial temporal lobe. The color bar denotes source amplitude. The shaded gray area denotes the source analysis time window.

**FIGURE 6:**
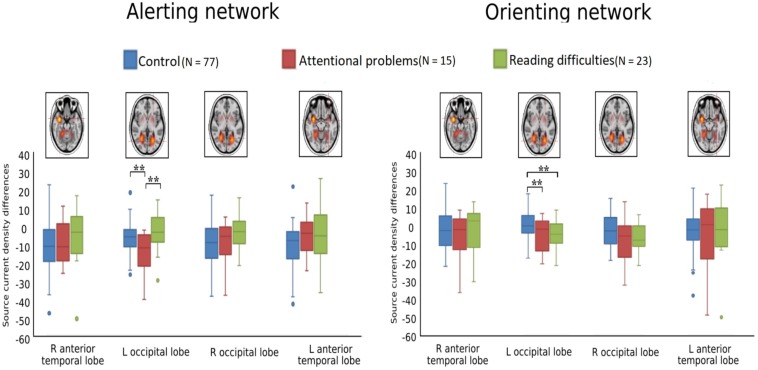
Boxplots of alerting (double-cued vs. non-cued target stimuli) and orienting (spatially cued vs. center-cued target stimuli) for source strength for control (blue, *N* = 77), attentional problems (red, *N* = 15), and reading difficulties (green, *N* = 23) groups; **p < 0.005. The FDR corrected alpha value is 0.012.

Figures 4, 5 show the grand-averaged source waveforms for all conditions between groups. The group main effect (Figure 6) was significant and showed a difference in the left occipital lobe for the alerting (double-cued vs. non-cued target stimuli) and orienting network (spatially cued vs. center-cued target stimuli).

Figure 6 and Table 3 show comparisons of control children and children with AP. The alerting network showed a significant main effect of condition in the left and right anterior temporal lobes and in the left and right occipital lobes. Increased activity resulting from the double-cued target stimulus as compared to a non-cued target stimulus was also observed. The interaction effect between condition and group was significant in the left occipital lobe, with the AP children having a larger alerting effect compared to the control children. The main effect of group was significant in the left occipital lobe of children with AP, as they exhibited larger responses than those of children in the control group.

**TABLE 3:** Repeated measures ANOVA test statistics for alerting, orienting, and inhibition sources between control (*N* = 77) and attentional problems (*N* = 15) children.

Control (*N* = 77) vs. AP (*N* = 15)																
**Alerting (double cue vs. no cue)**	**R anterior temporal lobe**				**L occipital lobe**			**R occipital lobe**			**L anterior temporal lobe**					
	**df**	***F***	***P***	**η^2^p**	***F***	***P***	**η^2^p**	***F***	***P***	**η^2^p**	***F***	***P***	**η^2^p**			
Main effect of condition	1	20.538	0.000	0.186	47.541	0.000	0.346	22.256	0.000	0.198	18.105	0.000	0.167			
Condition × group interaction	1	0.006	0.941	0.000	9.802	0.002	0.098	0.001	0.972	0.000	2.375	0.127	0.026			
Main effect of group	90	1.119	0.293	0.012	18.413	0.000	0.170	5.634	0.020	0.059	0.020	0.888	0.000			
**Orienting (spatial cue vs. center cue)**	**R anterior temporal lobe**				**L occipital lobe**			**R occipital lobe**			**L anterior temporal lobe**					
	**df**	***F***	***P***	**η^2^p**	***F***	***P***	**η^2^p**	***F***	***P***	**η^2^p**	***F***	***P***	**η^2^p**			
Main effect of condition	1	0.009	0.926	0.000	1.776	0.186	0.019	11.748	0.001	0.115	0.813	0.370	0.009			
Condition × group interaction	1	0.656	0.420	0.007	7.044	0.009	0.076	2.080	0.153	0.023	0.013	0.909	0.000			
Main effect of group	90	0.004	0.834	0.000	5.418	0.022	0.057	2.763	0.100	0.030	1.080	0.301	0.012			
**Inhibition (incongruent vs. congruent)**	**Medial prefrontal cortex**				**Medial frontal cortex**			**L anterior temporal lobe**			**R medial temporal lobe**			**L medial temporal lobe**		
	**df**	***F***	***P***	**η^2^p**	***F***	***P***	**η^2^p**	***F***	***P***	**η^2^p**	***F***	***P***	**η^2^p**	***F***	***P***	**η^2^p**
Main effect of condition	1	1.920	0.169	0.021	0.012	0.914	0.000	0.455	0.502	0.005	1.107	0.296	0.012	3.060	0.084	0.033
Condition × group interaction	1	0.662	0.418	0.007	5.006	0.028	0.053	0.579	0.451	0.006	1.363	0.246	0.015	4.697	0.033	0.050
Main effect of group	90	1.093	0.299	0.012	1.883	0.173	0.021	1.432	0.235	0.016	5.313	0.023	0.056	3.363	0.070	0.036

*AP, Children with attentional problems; L, left; R, Right; df, denotes degrees of freedom; η^2^p, partial eta-squared. The FDR corrected alpha value is 0.012.*

The orienting network showed a significant main effect of condition in the right occipital lobe with increased activity to the spatially cued target stimulus compared to the center-cued target stimulus. The interaction effect between condition and group in the left occipital lobe showed a significantly larger orienting effect in children with AP than in control children.

Figure 6 and Table 4 compare control children and children with RD. The alerting network showed a significant main effect for condition in the left and right anterior temporal lobes and in the left and right occipital lobes. There was also increased activity in response to the double-cued target stimulus compared to a non-cued target stimulus. The main effect of group was significant in the left occipital lobe with control children having larger responses than children with RD.

**TABLE 4:** Repeated measures ANOVA test statistics for alerting, orienting, and inhibition sources between control (*N* = 77) and reading difficulties children (*N* = 23).

Control (*N* = 77) vs. RD (*N* = 23)																
**Alerting (double cue vs. no cue)**	**R anterior temporal lobe**				**L occipital lobe**			**R occipital lobe**			**L anterior temporal lobe**					
	**df**	***F***	***P***	**η^2^p**	***F***	***P***	**η^2^p**	***F***	***P***	**η^2^p**	***F***	***P***	**η^2^p**			
Main effect of condition	1	14.043	0.000	0.125	8.105	0.005	0.076	15.222	0.000	0.134	17.684	0.000	0.153			
Condition × group interaction	1	2.112	0.149	0.021	2.337	0.130	0.023	2.811	0.097	0.028	4.638	0.034	0.045			
Main effect of group	98	0.571	0.452	0.006	8.129	0.005	0.077	1.498	0.224	0.015	2.757	0.100	0.027			
**Orienting (spatial cue vs. center cue)**	**R anterior temporal lobe**				**L occipital lobe**			**R occipital lobe**			**L anterior temporal lobe**					
	**df**	***F***	***P***	**η^2^p**	***F***	***P***	**η^2^p**	***F***	***P***	**η^2^p**	***F***	***P***	**η^2^p**			
Main effect of condition	1	5.473	0.021	0.053	1.954	0.165	0.020	12.234	0.001	0.111	2.774	0.099	0.028			
Condition × group interaction	1	1.578	0.212	0.016	8.345	0.005	0.078	2.266	0.135	0.023	0.645	0.424	0.007			
Main effect of group	98	0.242	0.624	0.002	6.720	0.011	0.064	2.821	0.096	0.028	1.315	0.254	0.013			
**Inhibition (incongruent vs. congruent)**	**Medial prefrontal cortex**				**Medial frontal cortex**			**L anterior temporal lobe**			**R medial temporal lobe**			**L medial temporal lobe**		
	**df**	***F***	***P***	**η^2^p**	***F***	***P***	**η^2^p**	***F***	***P***	**η^2^p**	***F***	***P***	**η^2^p**	***F***	***P***	**η^2^p**
Main effect of condition	1	0.073	0.787	0.001	1.558	0.215	0.016	1.900	0.171	0.019	4.604	0.034	0.045	0.844	0.361	0.009
Condition × group interaction	1	0.942	0.334	0.010	1.124	0.292	0.011	0.098	0.755	0.001	4.018	0.048	0.039	0.212	0.647	0.002
Main effect of group	98	0.018	0.893	0.000	3.824	0.053	0.038	0.007	0.933	0.000	6.563	0.012	0.063	5.454	0.022	0.053

*RD, Children with reading difficulties; L, left; R, Right; df, denotes degrees of freedom; η^2^p, partial eta-squared. The FDR corrected alpha value is 0.012.*

The orienting network showed a significant main effect for condition in the right occipital lobe. There was increased activity to the spatially cued target stimulus compared to a center-cued target stimulus. The interaction effect between condition and group in the left occipital lobe showed a significantly larger orienting effect in children with RD than in control children. The main effect of group was significant in the left occipital lobe with the control children having smaller responses than children with RD.

The inhibition network showed the main effect for group was significant in the right medial temporal lobe with children with RD showing a smaller response than children in the control group.

Figure 6 and Table 5 show comparisons of children with AP and children with RD. The alerting network showed a significant main effect for condition in the left and right anterior temporal lobes and the left and right occipital lobes. There was increased activity to the double-cued target stimulus compared to a non-cued target stimulus. The interaction effect between condition and group was significantly larger in the left occipital lobe of children with AP having a larger alerting effect than in children with RD.

**TABLE 5:** Repeated measures ANOVA test statistics for alerting, orienting, and inhibition sources between attentional problems (*N* = 15) and reading difficulties (*N* = 23) children.

AP (*N* = 15) vs. RD (*N* = 23)																
**Alerting (double cue vs. no cue)**	**R anterior temporal lobe**				**L occipital lobe**			**R occipital lobe**			**L anterior temporal lobe**					
	**df**	***F***	***P***	**η^2^p**	***F***	***P***	**η^2^p**	***F***	***P***	**η^2^p**	***F***	***P***	**η^2^p**			
Main effect of condition	1	7.255	0.011	0.168	15.343	0.000	0.299	8.652	0.006	0.194	2.880	0.098	0.074			
Condition × group interaction	1	1.013	0.231	0.027	9.741	0.004	0.213	1.643	0.208	0.044	0.098	0.757	0.003			
Main effect of group	36	0.097	0.757	0.003	2.905	0.097	0.075	1.831	0.184	0.048	1.487	0.231	0.040			
**Orienting (spatial cue vs. center cue)**	**R anterior temporal lobe**				**L occipital lobe**			**R occipital lobe**			**L anterior temporal lobe**					
	**df**	***F***	***P***	**η^2^p**	***F***	***P***	**η^2^p**	***F***	***P***	**η^2^p**	***F***	***P***	**η^2^p**			
Main effect of condition	1	0.785	0.381	0.021	7.674	0.009	0.176	9.117	0.005	0.203	0.799	0.377	0.022			
Condition × group interaction	1	2.086	0.157	0.055	0.004	0.947	0.000	0.001	0.972	0.000	0.114	0.737	0.003			
Main effect of group	36	0.035	0.853	0.001	0.037	0.848	0.001	0.036	0.851	0.001	0.003	0.954	0.000			
**Inhibition (incongruent vs. congruent)**	**Medial prefrontal cortex**				**Medial frontal cortex**			**L anterior temporal lobe**			**R medial temporal lobe**			**L medial temporal lobe**		
	**df**	***F***	***P***	**η^2^p**	***F***	***P***	**η^2^p**	***F***	***P***	**η^2^p**	***F***	***P***	**η^2^p**	***F***	***P***	**η^2^p**
Main effect of condition	1	0.262	0.612	0.007	0.653	0.424	0.018	0.148	0.703	0.004	0.235	0.631	0.006	0.912	0.346	0.025
Condition × group interaction	1	1.921	0.174	0.051	0.878	0.355	0.024	0.214	0.647	0.006	5.404	0.026	0.131	3.452	0.071	0.087
Main effect of group	36	0.873	0.356	0.024	0.135	0.716	0.004	1.098	0.302	0.030	0.002	0.962	0.000	0.025	0.874	0.001

*AP, Children with attentional problems; RD, Children with reading difficulties; L, left; R, Right; df, denotes degrees of freedom; η^2^p, partial eta-squared. The FDR corrected alpha value is 0.012.*

The orienting network showed a significant main effect of condition in the left and right occipital lobes with increased activity to the spatially cued target stimulus compared to the center-cued target stimulus.

The condition by group interactions for the repeated measures ANOVAs with trial numbers as covariates are significant. Alerting: *F*(2,110) = 6.685, *p* = 0.002, η_p_^2^ = 0.108. Orienting: *F*(2,110) = 6.865, *p* = 0.002, η_p_^2^ = 0.111.

## Discussion

We examined the reaction time performance, event-related potentials (ERP), and neuronal source activations of attentional sub-processes related to alerting, orienting, and inhibition using the attention network test (ANT) in typically developing 12−13-year-old children, as compared to those with attentional problems (AP) and those with reading difficulties (RD). Our results on reaction times (RT) showed that there were no significant differences in the reaction time performance for the alerting, orienting, and inhibition effects between any of the groups, although children with RD had slower RTs in general. The ERP sensor-level analyses did not reveal statistically significant differences in the target-related N1 or P3 between groups. However, neuronal source activity did show group differences (see Table 6). Children with AP showed a larger alerting effect (double-cued vs. non-cued target stimuli) in the left occipital lobe compared to control children and children with RD. Children in the control group showed a smaller orienting effect (spatially cued vs. center-cued target stimuli) in the left occipital lobe compared to children with AP and children with RD. No group differences were found for the neuronal sources related to the inhibition effect.

**TABLE 6:** Summary of neuronal source results related to alerting (non-cued vs. double-cued target stimuli), orienting (center-cued vs. spatially cued target stimuli), and inhibition (incongruent vs. congruent target stimuli) sub-processes of the attention network.

Sub-processes	Neuronal source	Group × condition effect	Direction of group difference
Alerting Source: DC vs. NC	L occipital lobe	C ≠ AP AP ≠ RD C = RD	C < AP RD < AP
Orienting Source: SC vs. CC	L occipital lobe	C ≠ AP C ≠ RD AP = RD	C < AP C < RD

*C, control children; AP, children with attentional problems and RD, children with reading difficulties; L, left; R, right; not equal (≠), interaction effect; equal (=), no interaction effect; NC, non-cued target stimuli; DC, double-cued target stimuli; CC, center-cued target stimuli; SC, spatially cued target stimuli.*

A meta-analysis as well as individual studies on children and adults with ADHD examining reaction times for the alerting effect (non-cued vs. double-cued target stimuli) and orienting effect (center-cued vs. spatially cued target stimuli) found no differences between control and ADHD groups in that regard (Berger and Posner, 2000; Huang-Pollock and Nigg, 2003; Adólfsdóttir et al., 2008; Kratz et al., 2011; Fabio and Urso, 2014). In line with these studies, our results on RT for alerting and orienting effect showed no group differences between controls and children with AP.

Previous studies on dyslexics (Bednarek et al., 2004; Goldfarb and Shaul, 2013) showed that there was a significant difference in the inhibition effect (incongruent target vs. congruent target) compared to a control group. In contrast with these studies, our results on RT for inhibition effects showed no group differences.

When examining brain activity using ERPs at the sensor level, a comparison of target-related N1 and P3 measures between control, AP, and RD groups did not show group differences in any of the three attention networks (alerting, orienting, or inhibition). To our knowledge, there have been no previous findings on target-related N1 amplitude modulation associated with alerting and orienting effects in children or adults with attentional or reading problems within the same study. There seem to be differences between the groups in the pre-stimulus (before target onset) time window. Future studies on N1- alerting and orienting effects during the pre-stimulus period could therefore reveal further processing differences between the groups.

In our investigation, children with AP did not differ from control and RD children with respect to P3 amplitude for the inhibition effect. This is in contrast to earlier studies, which found group differences in P3 in adults (Kratz et al., 2011; Hasler et al., 2016). Both of these studies showed a lower amplitude of P3 in the ADHD group compared to the control group, suggesting an ineffective attentional allocation to stimulus processing and evaluation. An adult study on lateralized ANT (the target being an arrow up or down and presented to the left or right of the fixation cross) supports our finding in the RD group that inhibition of irrelevant information measured by the P3 ERP component to the target (NoGo P3) is preserved in dyslexia (Mahé et al., 2014).

However, as described above, our RT results do not show group differences in alerting, orienting, and inhibition effects. It is possible that the scalp-level ERP may not be able to capture the differences in these attentional processes. One reason for this could be the use of cluster-based permutation statistics, which could yield results that are more conservative compared to some earlier studies (Maris and Oostenveld, 2007; Pernet et al., 2015). A statistically more sensitive method might have been the use of ANOVA for the selected set of electrodes, but this has the drawback of arbitrary channel selection not being the best representation of the actual brain responses. Therefore, we examined source-level information to disentangle the neural sources in the AP and RD groups utilizing the source model derived from the control group data (Santhana Gopalan et al., 2019).

The N1-related sources for the target stimulus were localized in the left and right occipital lobes and the left and right anterior temporal lobes between 140 ms and 200 ms. However, the left anterior temporal lobe did not show any alerting or orienting effect differing from zero and was therefore excluded from further analysis and interpretation. P3-related sources for the target stimulus were localized in the medial prefrontal cortex, medial frontal cortex, left anterior temporal lobe, and left and right medial temporal lobes.

There is evidence for structural and functional changes in the left occipital lobe (lingual gyrus) in the ADHD group compared to typically developing children (Dickstein et al., 2006; Xia et al., 2014; Lei et al., 2015). Furthermore, in an adult study, it was shown that shifting of attention from the cue to the target stimulus activates the occipital lobe (Corbetta et al., 1998). Our results in children with AP showed an increased neuronal response in the left occipital lobe for the alerting effect (double-cued vs. non-cued target stimuli) compared to control children. This could be interpreted as an atypical attentional visual process for the target stimulus based on warning cue information. Attentional disengagement and voluntary orienting have been considered important aspects of top-down attentional control processes related to selective sensory and motor processing (Hopfinger et al., 2000). It has been suggested that a network consisting of the occipital lobe, central, and parietal areas is involved in top-down attentional control, as evidenced by studies showing these areas to be active when following a cue to shift the spatial attention toward the target stimulus (Hopfinger and Ries, 2005; Corbetta et al., 2008; Zhao et al., 2017). Based on previous studies, children with AP who display larger orienting effect (center-cued vs. spatially cued target stimuli) than control group could be interpreted as having reduced top-down control (Corbetta et al., 2008; Zhao et al., 2017).

The neuronal source activation across both double-cued and non-cued target stimuli differed between children with AP and control children in the right occipital lobe and also between children with RD and control children in the left occipital lobe. This difference was not related to the alerting effect but instead to the target stimulus, regardless of which of the two cueing conditions was examined. This shows that children with AP and children with RD might have subtle differential processing atypicalities in the anticipation of a visual warning cue and in response preparation toward the target stimuli (Konrad et al., 2005, 2006; Xuan et al., 2016).

Our finding of the group difference between children with RD and controls for the alerting sub-processes in the left occipital lobe could be linked to structural and functional neuroimaging studies of dyslexia (Pugh et al., 2000; Démonet et al., 2004; Richlan, 2012; Xia et al., 2017). A recent review on developmental dyslexia has suggested that left posterior occipitotemporal dysfunction is a secondary deficit area in dyslexia, as it was assumed that phonological processing deficits reflected in the temporoparietal junction would lead to interference with the development of the left occipitotemporal cortex (Kronbichler and Kronbichler, 2018). Therefore, it is possible that atypical processing of visual information in the left occipital regions could be seen in children with RD, even for non-linguistic material.

With respect to the inhibition network, previous studies showed an abnormal activity pattern of the medial frontal region, including the anterior cingulate cortex (ACC) and parietal cortex compared to control groups (in children and adults) (Durston et al., 2003; Konrad et al., 2005, 2006). In contrast with the previous studies, our study showed no group differences in the neuronal sources related to the inhibition network. The non-correspondence between RT results and the neuronal source results may be due to that RT results represent the amount of differences in RT performance processes and that the processes assessed in this study could only respond to a few cognition attributes that mediate the task’s performance (Wilkinson and Halligan, 2004; Konrad et al., 2005).

The overall strength of the neural response does not reveal possible top-down or bottom-up modulation of the neural responses. Future studies should examine whether the frontal and temporal cortices interact during the inhibition effect and whether this interaction could partly explain the group differences observed. Connectivity analyses could reveal the direction of the effect between the regions, providing clues on whether the differences in temporal cortex activity are caused by top-down modulation from the frontal areas or whether the temporal cortex findings are independent of the activity in the frontal areas.

Generally, EEG/ERP source imaging has limitations in terms of spatial accuracy, making exact comparisons to fMRI studies difficult (Grech et al., 2008; Costa et al., 2015). It is also possible that some neuronal sources related to the AP and RD groups were not revealed when using this spatial filter source model, which was designed based on the control group as prior information for the activity during an ANT test. To overcome this limitation, neuronal source imaging could be carried out at an individual subject level and mapped to a corresponding MRI. It is also important to note that in this study, the number of participants in the attentional problems and reading difficulties groups were considerably smaller than for the control group. This limits the generalizability of the results and warrants further studies to verify the current findings.

In summary, both children with AP and children with RD showed differential results in alerting and orienting networks compared to control children with respect to the attention network task. Children with AP exhibited increased source activity in the left occipital lobe for the orienting effect. Furthermore, the children with RD showed different source activity in the left occipital lobe for the alerting and orienting networks. These results show how attentional processes differ across the attention network in children with AP and children with RD. This suggests different underlying mechanisms for attentional and reading problems. Overall, the results of reaction time performance and neuronal sources adds to the growing body of literature that has found the attention network to be a useful cognitive model for conceptualizing attentional problems and reading difficulties in children (Bednarek et al., 2004; Konrad et al., 2006; Booth et al., 2007; Mullane et al., 2011; Goldfarb and Shaul, 2013).

## Data Availability Statement

The analyzed data sets from this study are available from the research group upon request.

## Ethics Statement

The studies involving human participants were reviewed and approved by the ethics committee of the University of Jyväskylä, Finland. Written informed consent to participate in this study was provided by the participants’ legal guardian/next of kin.

## Author Contributions

OL, KL, JH, and PL designed the experiment. Research assistants, PS, OL, and PL recruited the participants and collected the data. PS analyzed the data, wrote the main manuscript, and created all figures. All authors commented on and reviewed the manuscript.

## Conflict of Interest

The authors declare that the research was conducted in the absence of any commercial or financial relationships that could be construed as a potential conflict of interest.
