# Supplementary material for: Attentional Processes in Children With Attentional Problems or Reading Difficulties as Revealed Using Brain Event-Related Potentials and Their Source Localization
Source: Front Hum Neurosci. 2020 May 8;14:160. doi: 10.3389/fnhum.2020.00160 (PMC7227392; doi:10.3389/fnhum.2020.00160)
Supplement: Supplementary file 1 [file Table_1.docx]

**Supplementary material**

**Supplementary Table 1.** Summary of rejected trials in proportion and their standard deviation for control (N=77), attentional problems (N=15), and reading difficulties (N=23) groups.

| Group | non-cued target stimuli | double-cued target stimuli | centre-cued target stimuli | spatially-cued target stimuli | congruent target stimuli | Incongruent target stimuli |
| --- | --- | --- | --- | --- | --- | --- |
| Control | 0.24 (0.131) | 0.20 (0.114) | 0.20 (0.110) | 0.21 (0.116) | 0.18 (0.102) | 0.15 (0.085) |
| AP | 0.29 (0.102) | 0.27 (0.116) | 0.25 (0.097) | 0.29 (0.089) | 0.23 (0.096) | 0.20 0.083) |
| RD | 0.32 (0.133) | 0.29 (0.116) | 0.25 (0.131) | 0.27 (0.113) | 0.23 (0.101) | 0.21 (0.100) |

Note: Control - control children; AP - children with attentional problems; RD - children with reading difficulties.

**Supplementary Table 2.** Mean (M) and standard deviation (SD) of residual variance for each condition and each group to determine the goodness of fit of the regional source model. No significant difference was found between any of the groups.

|  | Control | | AP | | RD | |
| --- | --- | --- | --- | --- | --- | --- |
|  | Mean | SD | Mean | SD | Mean | SD |
| no cue  double cue | 13.005  11.412 | 6.438  5.731 | 12.196  10.636 | 6.143  5.707 | 14.575  13.107 | 7.455  7.687 |
| center cue  spatial cue | 11.919  11.357 | 6.124  5.498 | 10.058  8.817 | 6.126  4.280 | 12.011  11.517 | 5.622  5.652 |
| Incongruent  Congruent | 15.512  14.818 | 7.594  7.672 | 14.533  13.881 | 7.064  8.617 | 16.778  15.650 | 8.388  7.532 |

Note: Values are in percentage. Control - control children; AP - children with attentional problems; RD - children with reading difficulties.

**Supplementary Table 3.** Summary of the reaction time (RT) results, accuracy and statistics for control (N=77), attentional problems (N=15), and reading difficulties (N=23) groups. Also shown is the RT (mean and standard deviation in millisecond) of each non-cued and cued target stimulus condition of the ANT.

| **Control group**  **(N = 77)**  **df (76)** | **Condition** | | **M (ms)** | **SD (ms)** | **Accuracy**  **M (SD)** | **Paired t-test for RT** | | | |
| --- | --- | --- | --- | --- | --- | --- | --- | --- | --- |
|  |  |  |  |  |  | **M^a^**  **(ms)** | **SD^a^ (ms)** | **t-value** | **Cohen’s D_z_** |
|  | Alerting | Non-cued | 785 | 99 | 0.98 (0.02) | 61.06 | 39.22 | 13.66*** | 1.61 |
|  |  | Double-cued | 724 | 87 | 0.97 (0.03) |  |  |  |  |
|  | Orienting | Centre-cued | 755 | 93 | 0.97 (0.04) | 50.73 | 45.31 | 9.82*** | 1.11 |
|  |  | Spatially-cued | 705 | 90 | 0.98 (0.02) |  |  |  |  |
|  | Inhibition | Incongruent | 806 | 97 | 0.96 (0.05) | 119.69 | 50.92 | 20.62*** | 2.35 |
|  |  | Congruent | 686 | 84 | 0.99 (0.01) |  |  |  |  |
| **Attentional problems**  **(N = 15)**  **df (14)** | Alerting | Non-cued | 790 | 66 | 0.98 (0.02) | 67.31 | 63.34 | 4.15** | 1.06 |
|  |  | Double-cued | 722 | 74 | 0.97 (0.02) |  |  |  |  |
|  | Orienting | Centre-cued | 745 | 62 | 0.98 (0.03) | 27.51 | 36.22 | 2.94* | 0.76 |
|  |  | Spatially-cued | 717 | 64 | 0.99 (0.02) |  |  |  |  |
|  | Inhibition | Incongruent | 810 | 71 | 0.97 (0.02) | 125.47 | 48.42 | 10.04*** | 2.59 |
|  |  | Congruent | 684 | 60 | 0.99 (0.01) |  |  |  |  |
| **Reading difficulties**  **(N = 23)**  **df (22)** | Alerting | Non-cued | 872 | 141 | 0.97 (0.03) | 79.40 | 40.97 | 9.29*** | 1.94 |
|  |  | Double-cued | 793 | 133 | 0.96 (0.05) |  |  |  |  |
|  | Orienting | Centre-cued | 847 | 139 | 0.97 (0.03) | 66.82 | 54.17 | 5.92*** | 1.23 |
|  |  | Spatially-cued | 780 | 149 | 0.97 (0.04) |  |  |  |  |
|  | Inhibition | Incongruent | 900 | 140 | 0.94 (0.05) | 144.17 | 39.81 | 17.37*** | 3.62 |
|  |  | Congruent | 756 | 128 | 0.99 (0.01) |  |  |  |  |

Note: ****p* < 0.0005, ***p* < 0.005, and **p* < 0.05 (two-tailed). M and SD denotes the mean and stand deviation. M^a^ and SD^a^ denote the average difference and standard deviation of the difference between the RTs for two target stimuli, respectively. The t-values denote test statistics with degrees of freedom (df). Cohen’s D_z_ denotes the effect size between RTs for different target stimuli.

**Supplementary Table 4.** A summary of independent sample t-tests between groups for reaction time effects.

|  | **Control vs. AP** | | | **Control vs. RD** | | | **AP vs. RD** | | |
| --- | --- | --- | --- | --- | --- | --- | --- | --- | --- |
| **Conditions** | **t-value**  **(df = 90)** | ***p-value*** | **Cohen’s d** | **t-value**  **(df = 98)** | ***p*-value** | **Cohen’s d** | **t-value**  **(df = 36)** | ***p*-value** | **Cohen’s d** |
| Non-cued  double cue  alerting (NC –DC) | -0.189  0.049  -0.505 | 0.850  0.961  0.615 | -0.053  0.014  -0.143 | -3.348  -2.921  -1.948 | 0.001  0.004  0.054 | -0.796  -0.694  -0.463 | -2.107  -1.859  -0.716 | 0.042  0.071  0.478 | -0.699  -0.617  -0.238 |
| Center-cued  spatial-cued  orienting (CC – SC) | 0.420  -0.512  1.870 | 0.675  0.609  0.065 | 0.119  -0.145  0.528 | -3.659  -2.981  -1.427 | 0.001  0.003  0.157 | -0.869  -0.708  -0.339 | -2.668  -1.536  -2.468 | 0.011  0.133  0.018 | -0.885  -0.510  -0.819 |
| Incongruent  congruent  inhibition (INCON – CON) | -0.147  0.082  -0.405 | 0.883  0.934  0.686 | -0.041  0.023  -0.114 | -3.647  -3.067  -2.117 | 0.001  0.002  0.037 | -0.867  -0.729  -0.503 | -2.297  -2.015  -1.299 | 0.027  0.051  0.202 | -0.762  -0.669  -0.431 |

Note: df denotes degrees of freedom. Cohen’s d denotes the effect size between groups. NC: non-cued target stimuli, DC: double-cued target stimuli, CC: center-cued target stimuli, SC: spatially-cued target stimuli, CON: congruent target stimuli, and INCON: incongruent target stimuli. The FDR corrected alpha value is 0.0120.
